# Loading Dose of Ceftazidime Needs to Be Increased in Critically Ill Patients: A Retrospective Study to Evaluate Recommended Loading Dose with Pharmacokinetic Modelling

**DOI:** 10.3390/antibiotics13080756

**Published:** 2024-08-11

**Authors:** Manon Launay, Edouard Ollier, Benjamin Kably, Félicien Le Louedec, Guillaume Thiery, Julien Lanoiselée, Sophie Perinel-Ragey

**Affiliations:** 1 Medical Intensive Care Unit, University Hospital of Saint-Etienne, 42000 Saint Etienne, France; 2 Pharmacovigilance Department, University Hospital of Saint-Etienne, 42000 Saint Etienne, France; 3 Mines Saint-Étienne, Université Jean Monnet, INSERM, U1059, SAINBIOSE, 42000 Saint Etienne, France; 4 Clinical Pharmacology Unit, University Hospital of Saint-Etienne, 42000 Saint Etienne, France; 5 Pharmacology Unit DMU BioPhyGen, European Hospital Georges-Pompidou, APHP, 75015 Paris, France; 6 Cancer Research Center of Toulouse (CRCT), Team 14, INSERM UMR1037, University of Toulouse, CS 53717, 31037 Toulouse, France; 7 Research on Healthcare Performance RESHAPE, INSERM U1290, Université Claude Bernard Lyon, 69008 Villeurbanne, France; 8 Service d’Anesthésie et de Réanimation Chirurgicale, CHU de Saint-Etienne, 42000 Saint Etienne, France

**Keywords:** ceftazidime, loading dose, critically ill patients, pharmacokinetics, volume of distribution, model-based simulations

## Abstract

To rapidly achieve ceftazidime target concentrations, a 2 g loading dose (LD) is recommended before continuous infusion, but its adequacy in critically ill patients, given their unique pharmacokinetics, needs investigation. This study included patients from six ICUs in Saint-Etienne and Paris, France, who received continuous ceftazidime infusion with plasma concentration measurements. Using MONOLIX and R, a pharmacokinetic (PK) model was developed, and the literature on ICU patient PK models was reviewed. Simulations calculated the LD needed to reach a 60 mg/L target concentration and assessed ceftazidime exposure for various regimens. Among 86 patients with 223 samples, ceftazidime PK was best described by a one-compartment model with glomerular filtration rate explaining clearance variability. Typical clearance and volume of distribution were 4.45 L/h and 88 L, respectively. The literature median volume of distribution was 37.2 L. Simulations indicated that an LD higher than 2 g was needed to achieve 60 mg/L in 80% of patients, with a median LD of 4.9 g. Our model showed a 4 g LD followed by 6 g/day infusion reached effective concentrations within 1 h, while a 2 g LD caused an 18 h delay in achieving target steady state.

## 1. Introduction

Ceftazidime (CAZ) is a broad-spectrum parenteral cephalosporin commonly used in septic, critically ill patients. The efficacy of β-lactams, such as ceftazidime, depends on the duration of nonprotein-bound plasma concentration above the minimum inhibitory concentration (%fT > MIC). A target concentration of free beta-lactam in plasma between four and eight times the minimum inhibitory concentration (MIC) of the causative bacteria for 100% of the dosing interval (fT ≥ 4–8 × MIC = 100%) has been proposed to maximize bacteriological and clinical response in intensive care unit (ICU) patients. For undocumented infections, the target range of ceftazidime blood concentration is therefore between 35 and 80 mg/L [1]. Because of its time-dependent bactericidal effect and 2 h half-life, CAZ is usually administered by continuous infusion [2]. Moreover, CAZ excretion is mainly renal and septic critical illness can lead to modified renal clearance (CL) [2].

Obese patients (20% of intensive care unit (ICU) patients [3]) are more likely to experience treatment failure and longer hospital length of stay than nonobese patients. Indeed, only 25–33% of obese patients achieve target concentrations with the standard dosing regimen, due to an increase in CAZ volume of distribution (Vd) and CL [4]. We have previously shown that CAZ plasma concentrations correlate with glomerular filtration rate (GFR) and body mass index (BMI) [5], and that obese patients with normal renal function should be administered 7–9 g/d by continuous infusion to reach the target range, rather than the usually recommended dose of 6 g/d [6].

In order to rapidly achieve target concentrations and bactericidal efficacy, the administration of a 2 g-CAZ loading dose (LD) is recommended before starting the continuous infusion. However, the adequacy of the LD usually administered should be investigated considering the special pharmacokinetic characteristics of ICU patients. Thus, the aim of this work was to develop a pharmacokinetic model of CAZ in septic critically ill patients, and perform simulations to evaluate drug exposure after administration of the recommended LD.

## 2. Results

A total of 86 patients (223 samples, with 1 to 9 samples per patient) were included in the original dataset to develop the population PK model; the majority of patients were male, with nearly 50% obese and adequate/normal value of GFR. (See Table 1). For the external validation dataset, 32 patients (32 samples) were included (See Table 1). BMI was only available for the model building dataset.

CAZ was best described by a one-compartment with additive error model and eGFR as a covariate on CL using allometric scaling (See Table 2). The typical CAZ CL was 4.45 L/h and Vd was 88 L.

**Figure 1:**
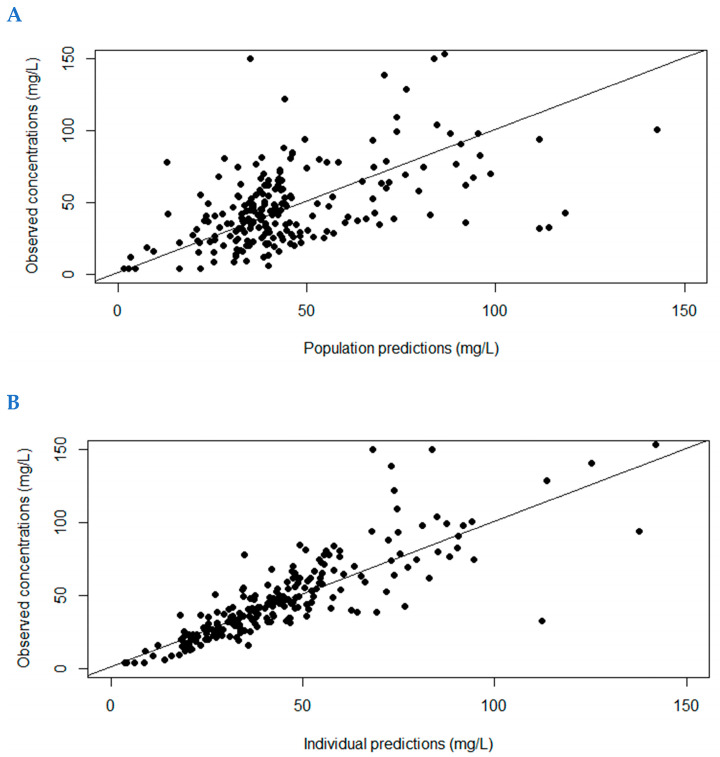
Observed versus population (**A**) and individual (**B**) predicted concentrations (mg/L) for the ceftazidime pharmacokinetic model.

Figure 1 shows the observed versus population (A) and individual predicted concentrations (B) for development dataset, and Figure S1 (Supplementary Data) shows the visual predictive checks. Figure S2 shows the observed versus population and individual predicted CAZ plasma concentrations for the validation dataset. All were considered as acceptable. MPDE and MDAPE for individual predictions were between −3.3 and 16.9%.

Median individual CAZ terminal half-lives was 11.9 h (IQR [8.8–18.4]). The predicted individual CAZ concentrations in the first 48 h after the start of treatment are shown in Figure S3. A delayed achievement of steady-state concentrations was observed when using a standard 2 g-LD and a continuous infusion with a median dosage of 6 g/d. The predicted individual CAZ concentrations were significantly lower at 24 h than at 48 h (34.6 ± 16.6 mg/L versus 43.7 ± 21.1 mg/L, *p* < 0.0001, paired *t*-test) for the 64 patients with available data and unchanged CAZ dose (see Table 3).

A total of 8 publications of PK models developed in ICU patients populations were reviewed [7,8,9,10,11,12,13,14], half of which considered the one-compartment model [7,8,9,10]. The median Vd was 37.2 L [min–max: 20.6–56.9]. Table 4 shows the simulated LD to achieve 60 mg/L in 80% of the patients from the models found in the literature. In all but one study, the simulated LD was higher than 2 g, the current recommended LD in the summary of product characteristics. Median LD was 4.9 g.

**Figure 2:**
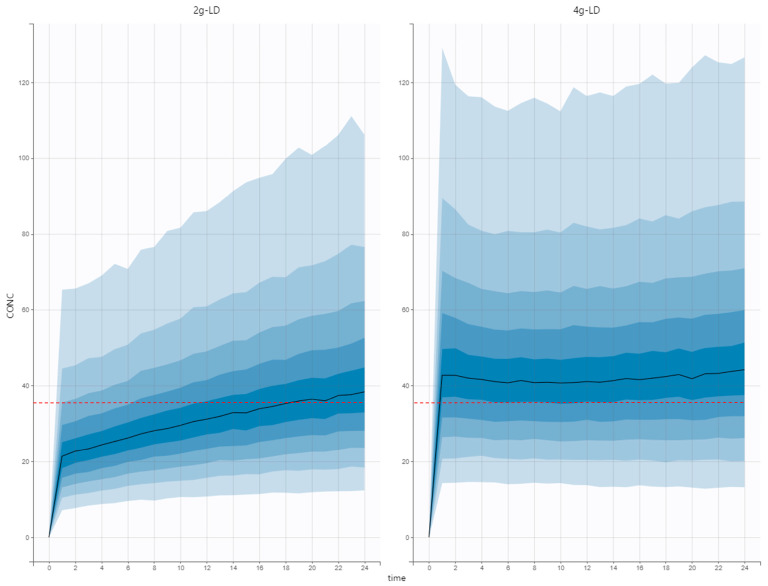
Results of Monte Carlo simulations with the standard regimen (2 g loading dose (LD) followed by 6 g of continuous infusion) on the left and the optimized loading dose regimen (4 g loading dose followed by 6 g of continuous infusion) on the right. Lower limit of the target range is shown using a red dotted line.

Simulations were conducted using our model based on the estimated pharmacokinetic parameters. The target concentration threshold of 35 mg/L was achieved within a median time of 1 h with a 4 g-loading dose immediately followed by a 6 g/d continuous infusion, compared to 18 h with a standard 2 g-loading dose (Figure 2).

## 3. Discussion

In critically ill patients receiving CAZ, a standard 2 g-LD seems insufficient to achieve the steady-state concentration threshold immediately after drug administration, probably because of the increased Vd observed in this particular population.

The PK model developed in this study fitted well the external validation cohort, with acceptable MPDE and MDAPE. In the specific context of ICU patients, Vd was 800% increased compared to noncritical nonobese patients [4]. Resuscitation fluids, positive inotropic agents, hypovolemia, hypoalbuminemia, hypotension, and organ dysfunction affect the pharmacokinetics of beta-lactams. Increased Vd has already been described in ICU patients, with values around 50–60 L, with an average BMI lower than what we observed in this study [7,10]. Surprisingly, contrary to our expectations, weight and BMI were not identified as significant covariates to explain Vd variability [5]. This could be due to the fact that almost 50% of the patients in our study population were obese, resulting in a higher mean volume of distribution with less variability.

Theoretically, an increase in Vd at a given CL would prolong the terminal half-life, possibly delaying the attainment of steady state [11]. In this study, the median CAZ half-life was actually six times longer than the usual half-life (11.9 h compared to 2 h [4]).Using an appropriate LD, steady-state plasma concentrations would be reached immediately. However, as illustrated in Figure 2, a 2 g-loading dose was inadequate for achieving target concentrations promptly in ICU patients. A delay of 18 h was indeed observed regarding our Monte Carlo simulations. The median CAZ individual predicted concentration at 24 h post treatment introduction was 34 mg/L. In other words, less than 50% of the patients would reach the lowest point of the recommended target range. According to the previously published models, the appropriate LD to achieve a plasma concentration of 60 mg/L would be 5 ± 2 g depending on the model chosen. For all but one PK study, simulated LD was higher than the currently recommended LD.

Delayed appropriate antibiotic treatment has been demonstrated to be associated with hospital mortality [15]. In acutely kidney-injured patients, there is growing evidence that unadjusted renal dosing of beta-lactam antibiotics in the first 24 to 48 h is warranted due to increased Vd and potential subtherapeutic levels [16]. But this strategy could led to overdosing with potential side effects. Potentially, increasing LD could resolve this issue by reaching the target quickly and using renal adjustment for continuous infusion after this LD. This point should be investigated in further prospective studies. Furthermore, using Monte Carlo simulations based on 18 patients treated with CAZ, *Delattre* et al. have shown that a higher first dose is recommended in critically ill patients (4 g for a 3 h infusion for a 96.5 probability of reaching the target), with the subsequent antibiotic dose administered 6 h later [17]. However, the current study is the first to investigate the optimal LD before continuous infusion in ICU patients.

Our study has several limitations. First, as CAZ plasma concentrations were coming from routine TDM, no early sample (<12 h after the administration of LD) was available and the estimation of Vd was therefore not optimal. Further studies should be conducted to properly document steady-state achievement in real-life settings. Second, the suggested LD of 4 g is theoretical and its safety has not been investigated. However, CAZ concentrations regarding our model-based simulations using this dosing regimen remained below the toxic threshold of 100 mg/L [18,19], and no antibiotic toxicity has been demonstrated in the literature with this dose.

## 4. Materials and Methods

### 4.1. Study Design

PK dataset for model development included all consecutive adult patients from a previously published study [6], treated with CAZ under continuous infusion after 2 g-LD and under therapeutic drug monitoring (TDM) evaluation [IRBN992021/CHUSTE].

### 4.2. Population

For model development cohort, this study enrolled all consecutive adult patients from six French ICUs (i.e., polyvalent, surgical, and nephrology ICUs of Saint Etienne University Hospital, polyvalent ICUs of Loire Private Hospital, Mutualiste Clinic, and Roanne Hospital) who received ceftazidime as a continuous infusion between 1 November 2019, and 31 October 2021, and underwent therapeutic drug monitoring (TDM) evaluation.

### 4.3. Blood Sampling

Ceftazidime TDM was routinely conducted based on the clinician’s discretion. Ceftazidime TDM for all six ICUs was performed at the Laboratory of Pharmacology, Toxicology, and Blood Gases of Saint Etienne University Hospital (France). The inclusion criteria required at least one total plasma concentration of ceftazidime collected at steady state, defined as being at least 6 h after the initiation of continuous infusion. The calibration range was 8 to 150 mg/L. Data lower than the lower limit of quantification were defined as 4 mg/L. The indicative threshold for CAZ was based on the usual target for steady-state plasma concentrations (35–80 mg/L) [1]. The external model validation dataset was obtained from data collected in ICU patients from Paris, France. The validation cohort study enrolled all consecutive adult patients from medical or surgical ICU who received ceftazidime as a continuous infusion between 1 May 2021, and 31 October 2022, and underwent TDM evaluation. Ceftazidime TDM was routinely performed at the Laboratory of Pharmacology of European Hospital Georges Pompidou in Paris (France). Patients with continuous renal replacement therapy were excluded.

### 4.4. PK Modelling and Evaluation

Data collected for covariate model development included age, sex, weight, height, status of ongoing COVID infection, BMI, serum creatinine at baseline and at CAZ sampling, protein concentration, CAZ regimen, and serum concentrations. GFR was estimated using the Chronic Kidney Disease–Epidemiology Collaboration (CKD-EPI) formula. Dosing and sampling times were used in the datasets to calculate elapsed time, and no assumptions were made about reaching steady state.

Data were analyzed with MONOLIX (MonolixSuite 2021 R1, Antony, France: Lixoft SAS, 2021). It was assumed that parameters of the model were lognormally distributed. The model was built stepwise, first identifying the best structural model and then evaluating the effects of covariates on CAZ plasma concentrations. Selection between competing models was statistically conducted using the likelihood-ratio test on objective function values (OFV), assuming a χ^2^ distribution for the OFV. A ΔOFV = −3.84 for one additional parameter was considered significant at alpha = 0.05, the significance level applied for both structural and stochastic components [20]. Additionally, parameter precision, covariance matrix condition number, residual goodness of fit, and prediction-corrected visual predictive checks (VPCs) were utilized for model selection and evaluation. The structural model identification involved assessing one- or two-compartment disposition, with interindividual variability examined in all relevant parameters. Following the establishment of an appropriate structural model, initial evaluation of parameter-covariate relationships was conducted using stepwise covariate modeling for covariates including body weight, body mass index, age, sex, protein concentration, serum creatinine, and creatinine clearance. The significance level for covariate incorporation was set at alpha = 0.05. The model has then been translated into R software (R version 3.2.3, R Foundation for Statistical Computing, Vienna, Austria) using the packages mapbayr and mrgsolve with the future goal of developing a shiny application accessible to a wider audience. External validation was performed by predicting CAZ concentrations for the validation dataset. To evaluate predictive performance of the model, goodness-of-fit plots were first examined. Then, the prediction error (PE) was calculated to evaluate the prediction performance of the model:

$$PE\;\left(\%\right)=\frac{C_{pred}-C_{obs}\;}{C_{obs}\;}$$

The model bias was determined using the median prediction error (MDPE) [21]. The inaccuracy of the model was determined using the median absolute prediction error (MDAPE). The model was considered acceptable if the MDPE was between ±20% and the MDAPE was ≤30% [21].

In order to investigate steady-state achievement, individual half-life were calculated, based on individual volume of distribution (${Vd}_{ind}$) and clearance (${CL}_{ind}$):

$${T_{1/2}}_{ind}=\frac{\ln\left(2\right)\times {Vd}_{ind}}{{CL}_{ind}}$$

Moreover, individual predictions at 24 ± 4 h and at 48 ± 4 h were compared using paired *t*-test for all patients with available data and unchanged CAZ amount.

A review of the literature was performed to search for pharmacokinetic (PK) models developed in ICU patients, in order to compare our results with existing models. Using parameters estimates of each PK models, the predicted LD to achieve the desired plasma CAZ target concentration (TC) was calculated for each published PK model [22]:

$$LD=Vd\times TC\times e^{{(\kappa \times \omega }_{V})}$$

In accordance with the published recommendations, 60 mg/L was considered to be an appropriate TC [9]. Parameter ω_V_ corresponds to the standard deviation of the random effects of the Vd and κ constant corresponds to a correction factor that allows interindividual variability to be taken into account [8]. The κ constant ensures that the loading dose is sufficient to achieve the target concentration for a specified proportion of patients. As random effects are assumed to follow a normal distribution, the constant κ represents the quantile of the normal distribution, encompassing the desired percentage of patients. For instance, if we aim for a loading dose adequate for at least 50% of patients, κ would be 0 (P(x < 0.5) = 0). If the target is a loading dose suitable for at least 80% of patients, κ would be 0.84 (P(x < 0.7995) = 0.84). This value is the one used in this study.

Finally, Monte Carlo simulations were performed using SimulX software 2021R1 (Lixoft, Orsay, France). The values of the pharmacokinetic parameters previously estimated were used to simulate the concentration profiles of 3000 patients following two dosing regimens: (i) 2 g of LD on day 1 followed by 6 g daily using continuous infusion (standard regimen) and (ii) 4 g of LD on day 1 followed by 6 g daily using continuous infusion) (optimized-LD regimen). The mean concentration and 90% confidence interval were determined for each regimen. Simulation endpoints was the delay to achieve a concentration higher than or equal to 35 mg/L (the minimal recommended concentration).

## 5. Conclusions

Despite current recommendations, the usually administered CAZ 2 g-LD seems insufficient in critically ill patients to achieve target steady-state concentrations immediately, probably because of the increased Vd observed in this particular population. Our results suggest to consider increasing to a 4 g-LD before starting continuous infusion. Considering these data, further prospective studies are needed to confirm this hypothesis in clinical practice, including from a safety point of view.

## Figures and Tables

**Table 1:** Demographics of patients included in the PK model development and validation. BMI = body mass index; GFR = glomerular filtration rate; NA = not available; ICU = intensive care unit.

	PK Model Development Dataset	External Validation Dataset
No. of patients/samples	86/223	32/32
Population	6 ICU (Saint-Etienne area)	1 ICU (Paris)
Age (years)	64.5 ± 11.9	NA
No. male	67 (77.9%)	23 (69.7%)
Weight (kg)	91.3 ± 25.3	77.4 ± 19.4
BMI (kg/m^2^)	31.2 ± 9.5 (47.7% obese patients)	NA
GFR (mL/min/1.73 m^2^)	87.6 ± 74.2	86.4 ± 40.2

**Table 2:** CAZ pharmacokinetic parameters. CL is clearance, Vd is the volume of distribution, GFR is the glomerular filtration rate estimated using the CKD-EPI formula expressed in mL/min/1.73 m^2^. The parameter $\beta_{GFR}^{CL}$ corresponds to the magnitude of the association between covariate and PK parameter. GFRmedian (i.e., 73.90 mL/min/1.73 m^2^) represents the median value of the covariates of the population. RSE is the relative standard error.

Parameter	Mean	RSE (%)	Shrinkage (%)
*Population*			
CL (L/h)	4.45	7.3	-
Vd (L)	88.0	18.3	-
*Covariate effect*			
$\mathrm{CL}\left(i\right)=\mathrm{CL}\left(pop\right){\times \left(\frac{GFRi}{GFRmedian}\right)}^{\beta_{GFR}^{CL}}\times e^{\eta_{CL}}$			
$\beta_{GFR}^{CL}$	0.9	15.5	-
*Interindividual variability (standard deviation)*			
CL (L/h)	0.46	11.5	38.9%
Vd (L)	0.57	25.6	82.5%
*Error model*			
Additive (mg/L)	0.39	6.2	-

**Table 3:** Ceftazidime individual predicted concentrations (mg/L) at 24 h and 48 h for all 64 patients with available data and unchanged ceftazidime amount.

CAZ Concentration	Individual Predictions at 24 h	Individual Predictions at 48 h
Min, mg/L	10.7	11.2
First quartile, mg/L	26.7	32.5
Median, mg/L	33.9	41.8
Mean, mg/L	34.6	43.7
Third quartile, mg/L	38.3	48.9
Max, mg/L	133.5	137.3
Target attainment, *n* (%)	27 (42.2)	41 (64.0)

**Table 4:** Simulated loading doses (LD) from published models in critically ill patients [7,8,9,10,11,12,13,14].

Reference	Type of Patients	Vd (L)	LD (g)
Current model	ICU adult patients	88	8.5
Werumeus Buning et al. [10]	ICU adult patients	48	5.3
Dailly et al. [11]	Burn adult patients	21	1.4
Falcone et al. [12]	Both ICU and non-ICU adult patients	26	3.4
Gómez et al. [10]	ICU adult patients	57	4.9
Georges et al. [11]	ICU adult patients	56	6.2
König et al. [15]	ICU patients receiving sustained low-efficiency dialysis	39	7.0
Li et al. [16]	Healthy volunteers and patients	20	2.4
Delattre et al. [14]	ICU adult patients	35	3.2

## Data Availability

The datasets used and/or analyzed in the current study are available from the corresponding author upon reasonable request.

## Supplementary Materials

The following supporting information can be downloaded at: https://www.mdpi.com/article/10.3390/antibiotics13080756/s1, Figure S1. Predicted corrected visual predictive checks for the CAZ pharmacokinetic model; Figure S2. Observed versus population (A) and individual (B) predicted ceftazidime concentration (mg/L) for the validation dataset; Figure S3. Ceftazidime individual predicted concentrations in the first 48 h showing the delayed achievement of steady-state concentrations.
