# Supplementary material for: Loading Dose of Ceftazidime Needs to Be Increased in Critically Ill Patients: A Retrospective Study to Evaluate Recommended Loading Dose with Pharmacokinetic Modelling
Source: Antibiotics (Basel). 2024 Aug 11;13(8):756. doi: 10.3390/antibiotics13080756 (PMC11350857; doi:10.3390/antibiotics13080756)
Supplement: Supplementary file 1 [file antibiotics-13-00756-s001.zip › antibiotics-3110444-supplementary.pdf]

Additional files

Figure S1. Predicted corrected visual predictive checks for the CAZ pharmacokinetic model.

The prediction intervals for each percentile are estimated for all simulated data and shown as coloured areas (pink for the 50th percentile and blue for the 10th and 90th percentiles). Outliers are highlighted by red dots and areas.

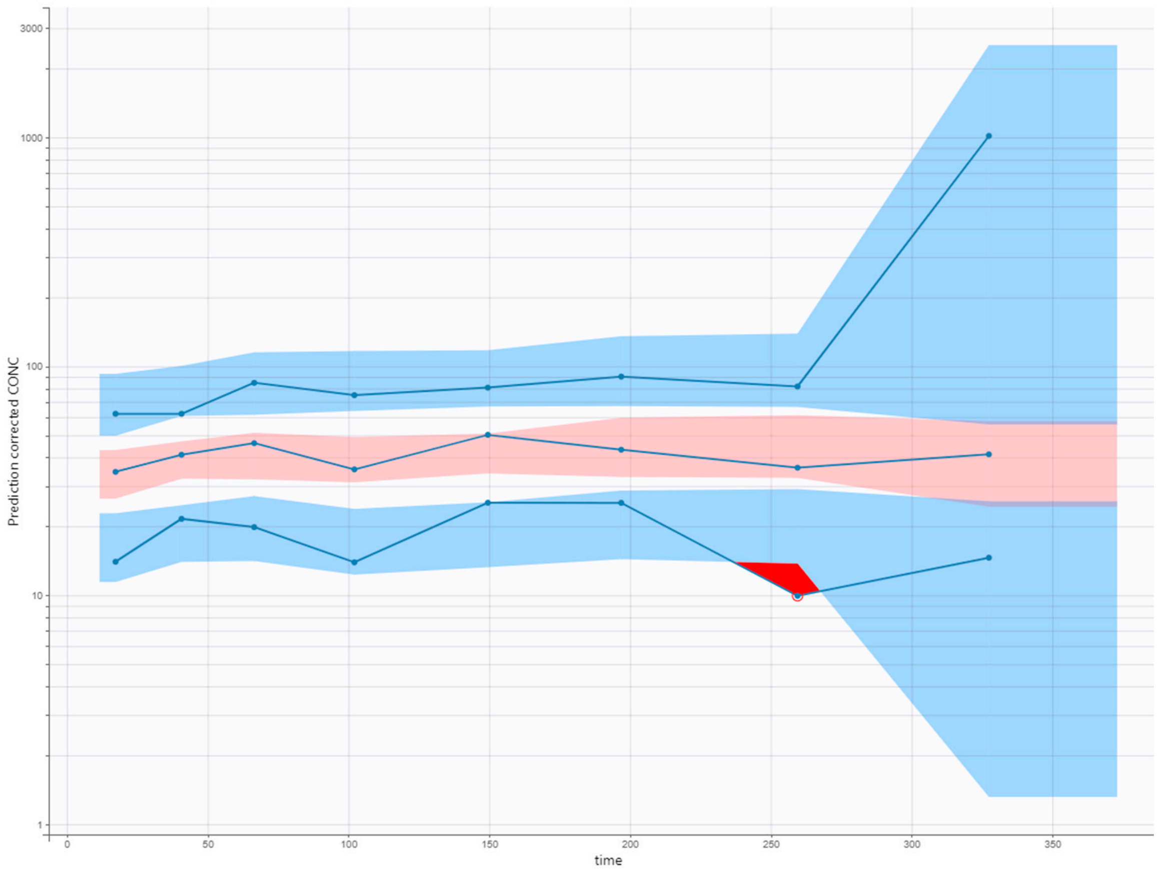

Figure S2. Observed versus population (A) and individual (B) predicted ceftazidime concentration (mg/L) for the validation data set.

Each black dot is a prediction.

A

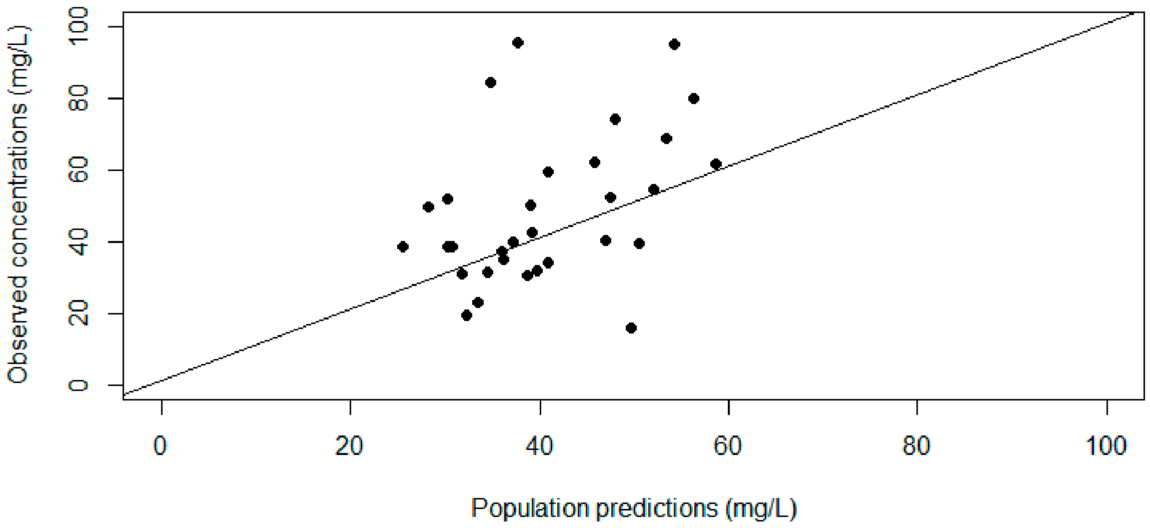

B

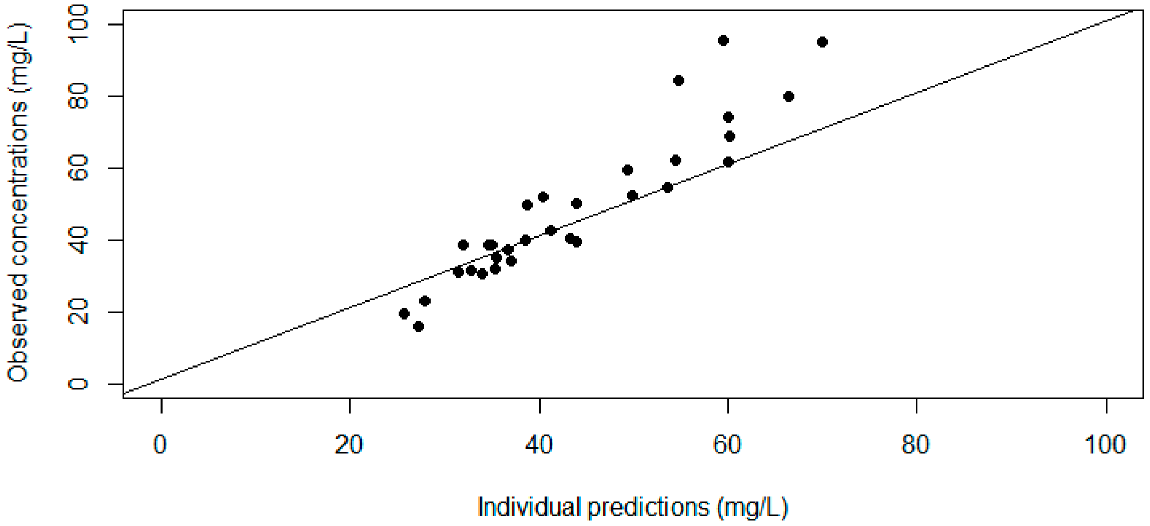

Figure S3. Ceftazidime individual predicted concentrations in the first 48h showing the delayed achievement of steady-state concentrations.

The target CAZ concentration was drawn using red dashed lines between 35 and 80 mg/L. Prediction for the same individual are indicated using black solid lines and dots.

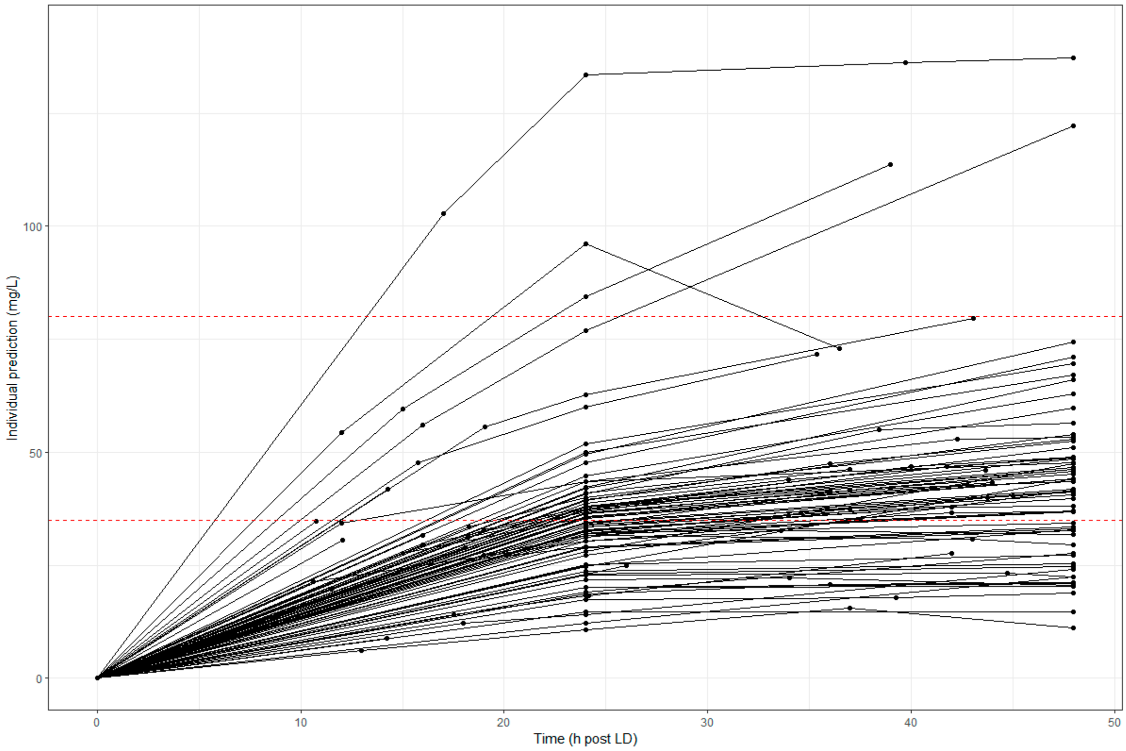
Individual prediction (mg/L)
100
50
0
0
10
20
30
40
50
Time (h post LD)
